# Latest Concepts in the Endodontic Management of Kidney Patients

**DOI:** 10.7759/cureus.54474

**Published:** 2024-02-19

**Authors:** Maryam Kuzekanani, Fatemeh Arabpour

**Affiliations:** 1 Endodontics, Endodontology Research Center, Kerman University of Medical Sciences and Health Services, Kerman, IRN; 2 Orthodontics, Shahid Sadoughi University of Medical Sciences, Yazd, IRN

**Keywords:** management, patients, kidney, endodontic, dental, considerations

## Abstract

Doubtlessly, kidney patients present a common challenge in endodontic practice, so specialists in this field should know and follow several key points regarding this group of medically compromised patients. This review paper aims to explain kidney disease and its complications, as well as notify and discuss the latest concepts on anesthesia, pain management, antibiotic prophylaxis/stewardship, and the risk of viral diseases for these patients, and also the oral manifestations of kidney diseases that may guide endodontists to diagnose kidney intervention and prevent hazardous consequences that may happen during or following endodontic practice on these patients. For this purpose, relevant keywords were searched on Scopus, PubMed, Medline, and Web of Science to find published papers from 1980 to July 2023. Based on the quality, validity, and novelty 57 published articles were selected to provide this review to notify the most important concepts and considerations regarding endodontic management of kidney patients. Overall, it is concluded that training and education of general dentists, as well as endodontic specialists with strong technical, scientific, human, and moral knowledge about kidney patients, with emphasis on the philosophy of prevention of common dangerous systemic consequences originating from endodontic treatments, is the responsibility of dental schools in undergraduate as well as post-graduate programs.

## Introduction and background

Kidney disease is one of the most common systemic disorders that can pose serious challenges to endodontists during root canal treatments. Nowadays, kidney patients have become more important in endodontic practice because of the increasing number of individuals who survive due to dialysis or kidney transplant after kidney failure. Therefore, informed and experienced endodontists can play an influential role in saving and preserving teeth and consequently improving the quality of life in this important group of medically compromised patients [1].

Kidney impairment causes high blood pressure, which can result in fatal cardiac conditions such as angina pectoris, ischemic heart diseases, myocardial infarction, as well as cerebrovascular diseases. The severity of kidney disease varies from primary to more advanced stages when the kidney completely fails and the patient needs dialysis or a kidney transplant to survive. Regarding this group of patients, if root canal treatment is needed, special care should be considered to avoid fatal complications [2-6].

Over time, chronic kidney disease (CKD) tends to progress to end-stage renal failure (ESRF). Although there are exceptions, attempts to stop or even slow down the progress of CKD have not been successful [7]. Chronic renal failure is a progressive disease characterized by the gradual destruction of nephrons and a consequent reduction of kidney function over months or years [2].

According to recent studies, apical periodontitis (AP) is significantly more prevalent in CKD patients. The association between the severity of AP and CKD markers suggests that AP may impact the progression of CKD, as well end-stage renal disease (ESRD) could alter the pathogenesis of AP. However, the findings of these studies do not establish a cause-and-effect interrelationship [8,9].

Patients with ESRD or those undergoing hemodialysis (HD) are at risk of developing several comorbidities, including hypertension, anemia, risk of bleeding, predisposing to infection, medication side effects, and oral manifestations associated with the disease itself or accompanied by HD treatment, which can pose challenges to both patient and the endodontist [10].

Endodontists must have enough information about this systemic condition and its challenges to improve the quality and outcome of root canal treatment for patients with this complication. In such patients, as mentioned, the risk of infection increases, and as a result, the treatment plan should aim to protect the patient from the potential risk of severe infection with dental origin. Also, due to the impaired immune system, there is an increased risk of bacterial endocarditis caused by dental infection in kidney patients [11].

Prevention of infection is essential because the most common cause of death in ESRD patients is cardiac arrest following infection and disease progression [10]. They are also at increased risk of blood loss because of the administration and use of heparin during dialysis, so they may be at risk for hemorrhage or even death following invasive dental procedures such as endodontic surgery, incision, or drainage [12].

Thus, an informed dentist or endodontist can play an influential role in preventing mortality as a result of disease complications. Of course, the clinician's role does not end here; because of changes in kidney function on blood purification, physicians and endodontists should consider appropriate drug administration according to the patient's condition [13].

In addition to the fact that 90% of patients experience oral symptoms, it has been noted that those who die from kidney failure have fewer teeth and more oral infections [14]. One study found that poor dental health was associated with early death in adults treated with HD, whereas preventive dental health practices were associated with prolonged survival. Moreover, the prevalence of AP is significantly higher in ESRD patients.

Considering the effect of AP on serum urea levels, AP treatment could be incorporated into the treatment plan for patients with ESRD. A recent study suggests that patients who receive RCT have a lower risk of death among dialysis patients, at the same time infectious diseases have a significant impact on the mortality rate among dialysis patients with non-RCT treated and periodontitis-involved teeth and as a result, appropriate treatments for dental problems may increase survival rates in them [8,9,15].

The role of endodontists becomes especially prominent here. Given the important complications of kidney patients, this review of the literature paper aims to discuss anesthesia and pain management, antibiotic prophylaxis and stewardship, risk of viral infections, and oral manifestations of kidney diseases in order to better manage dental and especially endodontic treatments and as a result, promote the quality and length of life for these patients.

## Review

Kidney disease and its complications

Patients with kidney disease experience several complications. High blood pressure plays a key role in the development of kidney failure. Hypertension can be both a cause and an effect of kidney failure. It is a common manifestation of kidney disease and is directly related to kidney dysfunction. More than one-third of kidney patients suffer from hypertension. The age group under 30 is at higher risk, also the prevalence of hypertension in female kidney patients is higher than in males, patients who have had kidney transplants are more likely to have high blood pressure than others [16].

High blood pressure can lead to heart complications such as myocardial infarction and congestive heart failure, and increase mortality rate [17]. That is why dentists must measure blood pressure before starting their work. Patients with ESRD, including stage 5 CKD, HD, and peritoneal dialysis (PD), are also at risk for bleeding because CKD increases the risk of atherothrombosis and is also associated with bleeding complications resulting from standard antithrombotic/antiplatelet therapy [18]. One study found that International Normalized Ratio (INR) increased in 30% of patients undergoing HD, with an INR between 1.5 and 3.5 and 12% rising over 3.5 [19]. This condition points to the necessity of being aware of the INR normal range and checking it before starting work (normal INR=1).

Anesthesia and pain management for patients with kidney disease

According to the latest instructions in the literature, articaine is the safest local anesthetic solution for patients with liver and kidney impairments [20]. Pain, most probably due to the use of the wrong type or dosage of analgesics, is common in patients with advanced CKD, and its management and control are essential. Poorly managed pain reduces the patient's quality of life. Non-steroidal anti-inflammatory drugs (NSAIDs) are considered very effective for managing various pains, including pain caused by inflammatory diseases, such as toothache [21]. At the same time, they may lead to various side effects, such as kidney poisoning. Long-term NSAIDs use may end in CKD [21,22]. Hence NSAIDs should be avoided in CKD patients [23]. Acetaminophen and aspirin are better choices for use in patients with advanced CKD stages 4-5 with no adverse effects on the progression of the condition [24].

Antibiotic prophylaxis for patients with kidney disease

The most recent antibiotic prophylaxis guideline currently available for patients with ESRD undergoing dental treatment was published by the American Heart Association (AHA) in 2003, so there is a need for a more adequate update and published protocol on this issue. According to the European Society of Endodontology statement on antibiotic prophylaxis in endodontics issued in 2018, in immunocompromised patients, including ESRD patients undergoing nonsurgical or surgical root canal treatment, especially apicoectomy clinicians should consider the following factors regarding antibiotic prescription.

*Infection status

*Complications related to infection

*Reaction to drugs

*In case of uncertainty, consultation with a physician is recommended before starting work [25].

Risk of viral disease for patients with kidney disease

Blood-borne viral diseases are the leading causes of death and disease in HD patients [26]. The prevalence rates of hepatitis C virus (HCV) infections are higher in patients with CKD, especially those undergoing maintenance dialysis, compared to the general population [27]. Untreated HCV infection has been associated with a more significant decline in kidney function in stages 3-5 CKD patients [28]. Acute kidney injury (AKI) and CKD are also more common in HIV patients than in the general population [29]. Therefore, it is necessary for the dentist to be aware of this issue and to have a proper attitude and approach to it.

Oral manifestations in kidney patients

These patients show a series of oral manifestations. Previous studies have demonstrated excessive rates of oral pathology in CKD patients with one or more oral complications, such as periodontitis, xerostomia, mucositis, and enamel hypoplasia [30-33]. In patients with CKD, stimulatory and non-stimulatory salivary flow rates decrease. The reduced salivary flow has been associated with oral lesions in most CKD patients (80%), and the most common finding is tasting abnormalities [34].

Periodontal disease is a chronic inflammation induced by various pathogens, and its frequency and severity are higher in patients undergoing dialysis than in healthy individuals [35]. Many studies have reported that more than half of HD patients exhibit periodontitis [36-39]. Similar to periodontal inflammation, microbial infection is prevalent in patients with CKD and ESRD in the root canal system and the periapical area [40]. Persistent root canal infections, such as AP, may contribute to microorganisms and inflammatory mediators invading the dentinal tubules, and accessory canals and damaging periapical tissue [41].

HD and reduced oral fluid intake can reduce salivary flow and lead to changes in the oral mucosa, xerostomia, and plaque formation, leading to an increase in the risk of microbial infection [42]. Chronic inflammation is thought to lead to several complications, including atherosclerosis, osteoporosis, fragility, diabetes, malignancy, etc. In addition, inflammatory and oxidative stress responses may worsen endothelial dysfunction in ESRD patients, accelerate coronary plaque formation, and increase cardiovascular risk [43-45].

A recent study conducted in 2019 showed that infectious disease was the leading cause of death in these patients. They found that more people who had not undergone RCT died from infectious diseases than those who had undergone RCT [41]. Hence, cardiovascular and cerebrovascular complications originating from infection in kidney patients are important concerns that should be better managed and prevented.

Discussions

Doubtlessly, kidney disease is one of the systemic dysfunctions that can pose challenges in endodontic practice especially in the elderly. Estimates of the global burden of disease indicate that kidney and urinary tract diseases account for approximately 830,000 deaths and 18,467,000 disability-adjusted life years annually, ranking 12th among causes of death and 1.4% of the total deaths, and 17th among the causes of disability. Kidney disease has become important in dentistry due to the increasing number of patients who survive kidney failure due to dialysis or kidney transplantation.

Consequences of kidney disease that affect dental management include pre-dialysis heparin injection, possible transmission of hepatitis B or C due to dialysis in public medical centers, secondary hyperparathyroidism, immunosuppressive therapy for nephritis syndrome, or transplant patients, medication-induced oral lesions, dose adjustment or contraindications of many drugs. Two examples of such problems are cephalosporins and tetracyclines and oral lesions seen in chronic renal failure [46].

High blood pressure is a common manifestation of kidney disease and is directly related to kidney impairment, which is also a significant cause of cardiac stroke, the leading cause of death in kidney failure; hence, endodontists and other dental clinicians, by checking blood pressure of these patients in clinic, should ensure that this important status of kidney patients is in the optimal range to prevent further complications [47]. The position of the chair does not require special consideration unless kidney disease is associated with another complication, because these patients are associated with bleeding problems, tests such as PFA-100 (platelet function analyzer) and platelet counts should be performed [3,13].

Since kidney function is impaired for blood purification, physicians and endodontists should consider appropriate drug administration [13]. Change in drug dosage is not limited to those who are on HD and considers other minor kidney interventions as well. Dental treatment is best done the day after dialysis when the patient's blood is well purified of toxins [48].

NSAIDs should not be used in patients with CKD [23]. However, the use of acetaminophen is safe for patients in advanced stages of (4-5) CKD without adverse effects on disease progression. CNS-depressant drugs such as chloral hydrate, though metabolized in the liver and red blood cells, are contraindicated for CKD patients [3,24].

Antibiotic prophylaxis, with penicillin and its derivatives, including vancomycin, clindamycin, and cephalosporins, is usually recommended prior to invasive root canal therapy in kidney patients. Although tetracycline, aminoglycosides, and antipyretic antibiotics, which are metabolized by the kidney, are contraindicated for these patients, they are recommended before invasive dental procedures [49].

This recommendation is controversial with the guidelines of the British Society for Antibiotic Prophylaxis; however, antibiotic prophylaxis is indicated during incision and drainage [3,13]. In a study conducted by Newadkar et al. on dental students regarding their knowledge and attitudes about oral manifestations of kidney patients, the students' knowledge was good [50].

Another study conducted by Caliento et al. on dentists regarding their knowledge and attitudes about the treatment of patients with kidney transplantation concluded that dentists are unfamiliar with how to use antibiotic prophylaxis for these patients which recommends the need to do further studies on the role and application of antibiotic prophylaxis and prescriptions to manage kidney patients in endodontic practice, as well as considering antibiotic resistance issues. Secondary hyperparathyroidism is one of the disorders seen in kidney patients [46,51].

It causes a series of radiolucent lesions in the jawbones that can mimic the appearance of an endodontic lesion. In cases where kidney disease is not associated with another disease, such as high blood pressure, there is no need to change the allowed number of anesthetic solution cartridges because these solutions are metabolized in the liver. The normal value of INR is 1, and if its rate reaches above 3.5, excessive bleeding can occur due to invasive dental and surgical procedures. Unlike HD, PD does not cause any additional problems in dental procedures [3].

The arterial-venous shunt concern should not be overlooked, and the arm with the shunt should never be used for intravenous or intramuscular injections [52]. The coexistence of diabetes and cardiovascular disease is more common in people on dialysis which makes the need to obey special considerations on these medical statuses as well. Gingival enlargement is caused by immunosuppressive drugs such as cyclosporine in those who have had a kidney transplant [3,53].

The training and education of endodontic specialists with strong technical, scientific, human, and moral knowledge to promote health and emphasize the philosophy of prevention of common systemic disease complications is the responsibility of dental schools. It must be ensured that endodontics postgraduate students and general dental students have the relevant basic knowledge in the prevention and early detection of various oral manifestations of systemic disorders, including kidney disease. Given that the clinicians, knowledge, and attitudes change over time, continuing educational courses can effectively promote the knowledge, attitude, and performance of endodontists [50,54-57].

## Conclusions

The most important consideration in the endodontic management of kidney patients is to check the blood pressure before starting the treatment. Articaine is the safest anesthetic solution, NSAIDs should not be prescribed, and acetaminophen and aspirin are the best analgesics for kidney patients. Blood-borne viral diseases are prevalent in dialysis kidney patients and this issue necessitates special self-care considerations for dental staff to prevent cross contaminations. A nephrologist consult is recommended for prescribing antibiotics and specific drugs because of kidney and probably liver drug clearance impairments. Overall, training and education of general dentists, as well as endodontic specialists with strong technical, scientific, human, and moral knowledge about kidney patients, emphasizing the philosophy of prevention of common dangerous systemic consequences originating from endodontic treatments is the responsibility of dental schools in undergraduate as well as post-graduate programs.
